# Counting Bites With Bits: Expert Workshop Addressing Calorie and Macronutrient Intake Monitoring

**DOI:** 10.2196/14904

**Published:** 2019-12-04

**Authors:** Nabil Alshurafa, Annie Wen Lin, Fengqing Zhu, Roozbeh Ghaffari, Josiah Hester, Edward Delp, John Rogers, Bonnie Spring

**Affiliations:** 1 Department of Preventive Medicine Northwestern University Feinberg School of Medicine Chicago, IL United States; 2 Department of Computer Science Northwestern University School of Engineering Evanston, IL United States; 3 Department of Electrical and Computer Engineering Northwestern University McCormick School of Engineering Evanston, IL United States; 4 School of Electrical and Computer Engineering Purdue University West Lafayette, IN United States; 5 Department of Materials Science and Engineering Northwestern University McCormick School of Engineering Evanston, IL United States; 6 Department of Biomedical Engineering Northwestern University McCormick School of Engineering Evanston, IL United States

**Keywords:** computer vision systems, computing methodologies, diet, energy intake, eating, eHealth, feeding behavior, mHealth, nutritional status, obesity, wearable technology

## Abstract

**Background:**

Conventional diet assessment approaches such as the 24-hour self-reported recall are burdensome, suffer from recall bias, and are inaccurate in estimating energy intake. Wearable sensor technology, coupled with advanced algorithms, is increasingly showing promise in its ability to capture behaviors that provide useful information for estimating calorie and macronutrient intake.

**Objective:**

This paper aimed to summarize current technological approaches to monitoring energy intake on the basis of expert opinion from a workshop panel and to make recommendations to advance technology and algorithms to improve estimation of energy expenditure.

**Methods:**

A 1-day invitational workshop sponsored by the National Science Foundation was held at Northwestern University. A total of 30 participants, including population health researchers, engineers, and intervention developers, from 6 universities and the National Institutes of Health participated in a panel discussing the state of evidence with regard to monitoring calorie intake and eating behaviors.

**Results:**

Calorie monitoring using technological approaches can be characterized into 3 domains: (1) image-based sensing (eg, wearable and smartphone-based cameras combined with machine learning algorithms); (2) eating action unit (EAU) sensors (eg, to measure feeding gesture and chewing rate); and (3) biochemical measures (eg, serum and plasma metabolite concentrations). We discussed how each domain functions, provided examples of promising solutions, and highlighted potential challenges and opportunities in each domain. Image-based sensor research requires improved ground truth (context and known information about the foods), accurate food image segmentation and recognition algorithms, and reliable methods of estimating portion size. EAU-based domain research is limited by the understanding of when their systems (device and inference algorithm) succeed and fail, need for privacy-protecting methods of capturing ground truth, and uncertainty in food categorization. Although an exciting novel technology, the challenges of biochemical sensing range from a lack of adaptability to environmental effects (eg, temperature change) and mechanical impact, instability of wearable sensor performance over time, and single-use design.

**Conclusions:**

Conventional approaches to calorie monitoring rely predominantly on self-reports. These approaches can gain contextual information from image-based and EAU-based domains that can map automatically captured food images to a food database and detect proxies that correlate with food volume and caloric intake. Although the continued development of advanced machine learning techniques will advance the accuracy of such wearables, biochemical sensing provides an electrochemical analysis of sweat using soft bioelectronics on human skin, enabling noninvasive measures of chemical compounds that provide insight into the digestive and endocrine systems. Future computing-based researchers should focus on reducing the burden of wearable sensors, aligning data across multiple devices, automating methods of data annotation, increasing rigor in studying system acceptability, increasing battery lifetime, and rigorously testing validity of the measure. Such research requires moving promising technological solutions from the controlled laboratory setting to the field.

## Introduction

The marked rise in obesity, particularly in the United States, is a complex sociodemographic and public health problem that is largely driven by poor diet, excessive caloric intake, and insufficient caloric expenditure [1]. Weight loss interventions in clinical and research settings have sought to curb this growing health concern by providing recommendations on decreasing caloric intake and increasing caloric expenditure [2]. Thus, subjective diet assessments—including food records, 24-hour dietary recall, and food frequency questionnaires (FFQs)—are often used in weight loss interventions to evaluate diet adherence and behavior change, although they are burdensome and prone to biased measurements of dietary intake and physical activity [3,4]. There is increasing interest in using health and fitness wearable devices to measure eating behaviors as they address the limitations of subjective diet assessments; these devices are set to become a US $48.2 billion market by 2023 [5]. Broad deployment of wearable activity trackers and heart rate monitors in the last decade has coincided with the need to reduce errors and improve our understanding of diet behaviors, calorie count, and nutrient intake. Using wearable technologies not only improves our understanding of diet behaviors but also aids the design of novel interventions to prevent overeating. Although emerging data suggest that diet and exercise programs are more successful at obtaining weight loss and healthy behavior change when they are mobile health (mHealth)–based interventions (ie, delivered via a mobile phone) compared with non-mHealth interventions (controls) [6], the effectiveness of combining mHealth-based interventions with wearable technologies to produce dietary change has yet to be properly studied.

To date, 3 types of technology-enabled wearable domains for calorie and nutrient monitoring have emerged: (1) *image-sensing technology* (eg, cameras coupled with novel algorithms that detect and analyze foods in an image using a food database); (2) *eating action unit (EAU)–based technology* (eg, wrist-worn sensors to capture eating and diet behaviors); and (3) *biochemical measures* (eg, sweat-sensing wearable technology that measures nutrient status). Although their impact on improving care and health outcomes remains untested, the validity of such devices is a prominent concern among researchers. Prior narrative reviews and surveys have focused on describing existing technologies [7-9] and algorithms [10], along with advantages and disadvantages of each type of wearable. Here, we have described the outcomes of a 1-day invitational workshop that identified challenges in developing technology-enabled, automated calorie-monitoring methods and proposed opportunities for future computing research in this field. We have also discussed how technology and objective measurements can support conventional subjective diet assessment approaches.

## Methods

An expert, consensus-building 1-day workshop, supported by the National Science Foundation and organized by Northwestern University, was held on June 20, 2017, in Chicago, Illinois. The *primary aim* of the workshop was to discuss the development, evaluation, and use of technology to detect and understand diet behaviors and estimate calorie and macronutrient intake.

A total of 30 participants from 6 universities and from the National Institutes of Health were selected to participate in the workshop. To capture varying perspectives across multiple fields, participants included population health researchers, such as behavioral scientists, nutritionists, obesity epidemiologists, and intervention developers (Bonnie Spring, Lisa Neff, Kevin Hall, and Marilyn Cornelius); computer scientists (Nabil Alshurafa, Adam Hoover, Edward Delp, and Mingui Sun); and engineers in biomedical, material science, and computer technology (Roozbeh Ghaffari, John Rogers, Veena Misra, Adam Hauke, Andrew Jajack, and Jason Heikenfeld). Multimedia Appendix 1 provides a list of participants at the workshop. Owing to the exploratory nature of this workshop, the organizers did not apply a theoretical framework.

A team consisting of at least 2 participants was organized to lead a discussion about one of the following topics: types of technology-enabled calorie and macronutrient monitoring, potential research gaps and technical challenges to advance the capture of energy expenditure, and methods for how technology can assist conventional subjective diet assessments. Workshop participants were also randomly separated into 2 groups to delineate key topics for future research. Overall, there was consensus regarding the need to refine technology-supported calorie- and macronutrient-monitoring approaches. The *primary deliverable* was a set of presentations delineating current gold standards for measuring energy intake and an appraisal of the state of research related to calorie- and macronutrient-monitoring technology. Experts within each technology-enabled wearable domain identified new insights and opportunities from these presentations and conversations, which were used to inform the final recommendations presented in this paper. A final review of the recommendations was performed by the authors of this paper.

## Technology-Enabled Domains for Measuring Calorie and Macronutrient Intake

**Figure 1:**
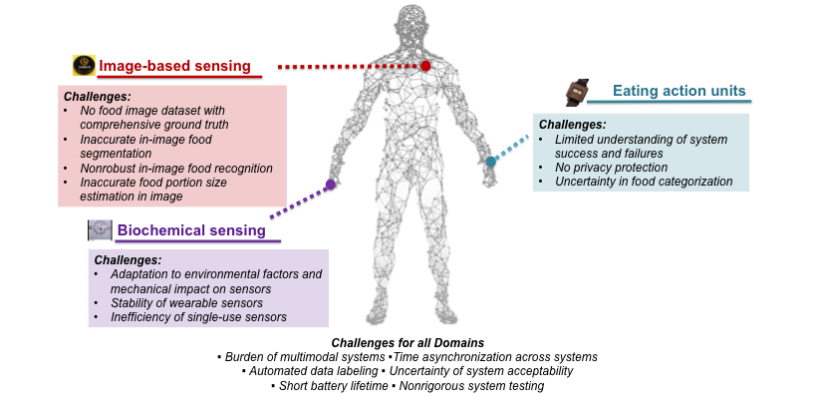
Overview of challenges in developing technology-enabled, automated caloric-monitoring methods.

Technology-enabled measures can reduce participant burden and increase granularity of diet data collection through automated measures [11]. We have explained each technology-enabled domain and identified key challenges to advancing the technology in the sections that follow (summarized in Figure 1). Each section also highlights potential research opportunities to advance technology-enabled devices in measuring calorie and macronutrient intake.

### Image-Based Sensing

Image-based sensing systems [12,13] that combine wearable or smartphone-based cameras with advanced computational machine learning models, in particular deep learning [14], have the capability to identify pixels in an image that represent foods (this is known as food segmentation), provide accurate timestamps when a meal is consumed (this is known as the metadata associated with the image), estimate consumption duration and frequency, and ascertain geographic eating locations (these 2 are commonly referred to as contextual information of the eating event). To enable these functions, researchers have focused on using image-based systems to identify food types [15]. These systems combine image-processing techniques and big data analytics to estimate energy contents for a meal [16,17] from food and nutrient databases, such as the Nutrition Data System for Research and the Food and Nutrient Database for Dietary Studies (FNDDS) [18]. Thus, image-based systems are unique as they use known foods in a database to guide the estimation of calorie intake and can provide a fairly accurate analysis of the consumed food types. However, accurate estimation of energy and nutrients in an image relies on the system’s ability to distinguish foods from the image background and to identify (or label) food items. Although there are promising advancements, several challenges remain in automating the estimation of calorie intake from cameras (Table 1).

**Table 1:** Challenges and research opportunities in adopting image-based sensing methods.

Challenge	Research opportunity
Lack of publicly available large-scale food image datasets with comprehensive *ground truth* labels	Develop feasible method to annotate food images crawled from the Web or collected from nutrition studies that can scale up
Inaccurate food image–segmentation algorithms	Reduce the burden of requiring fine-grain pixel-level training data for image segmentation and leverage accurate image or specific image region level information to improve food segmentation performance
Nonrobust food image–recognition systems	Design deep neural network–based models to capture structures in the image that are associated with specific foods and incorporate contextual information to improve robustness
Inaccurate food portion size estimation in image	Develop methods that can directly link food images to portion size; explore 3-dimensional information from newer camera sensors on mobile devices

#### Challenge: Lack of Publicly Available Large-Scale Food Image Datasets With Comprehensive Ground Truth Labels

A *ground truth* label, derived from observable data, is the objective verification of particular properties of a digital image, used to test the accuracy of automated image analysis processes. The ground truth of food images includes known, fixed features such as pixels representing food objects in the image (used for food segmentation), food types (used for food recognition), and food portion size in grams (used for portion size estimation). Such information is necessary to train the image analysis system to accurately estimate calorie intake from the image. Several publicly available food datasets contain a substantial amount of food images [18] and provide general labels of different food types, but none provide information relative to portion size, segmented food items, or additional context of the image source. To reliably build a machine learning model that automatically maps images to calories, successful image-based systems need large collections of food images (ie, food image datasets) with the necessary *ground truth* labels to improve the learned models. These *ground truth* labels should clearly delineate different food items even if they are on a single plate and should include portion size information for each food item.

##### Research Opportunities

To address the need for constructing large-scale food datasets with food images that provide comprehensive *ground truth* information, a solution is to merge food images sourced from the internet or from nutrition studies with manual annotation from crowd-sourcing platforms. Amazon Mechanical Turk (AMT) has been used for food image collection and annotation tasks [19,20], although AMT is not tailored for building large food image datasets efficiently with proper labels. This inefficiency may be partly attributed to its high cost and dependency on crowdsource workers unfamiliar with the context in which the data were collected (eg, restaurant food vs homemade meal). There are opportunities to develop novel tools that not only label foods in the image but also remove irrelevant images to aid crowdsource workers in accurately labeling necessary data. These approaches can be developed using a combination of crowd input and advanced automatic image analysis techniques [21].

#### Challenge: Inaccurate Food Image–Segmentation Algorithms

Image segmentation is the process of partitioning an image using an algorithm into disjointed and coherent regions on the basis of prespecified features. Food image segmentation is important for multifood images in which subsequent analysis, such as recognition and portion size estimation, depends on having accurate segmentation of each food in the image. Owing to the complexity of food images (eg, occlusion, hidden or mixed foods, and shadows), accurate food image segmentation is a difficult task and affects the ability of image-based sensing systems to identify food types. Previous studies [22-26] have used image-segmentation methods such as contour-to-region, graph-based, and superpixel-based approaches. A segmentation method based on deep neural networks has been proposed [27] to reliably build a model that automatically segments foods. However, these models require pixel-level food labels or labeled bounding boxes to indicate regions containing foods, which is time-consuming and computationally expensive.

##### Research Opportunities

Owing to the inefficiency and high expense, some studies have applied graph-based methods to select regions containing foods [28] or have explored techniques where only image-level labels indicating the presence or absence of foods are required instead of requiring pixel-level labels of food objects [29]. The opportunity to advance such methods remains, as does the main opportunity of creating efficient segmentation algorithms that provide pixel-level labels and training for each image in a food dataset.

#### Challenge: Nonrobust Food Image Recognition Systems

Research [30-32] in food recognition has analyzed multiple features and classification algorithms (aimed at identifying foods) that are effective but mainly restricted to a known food dataset that has been established a priori. Researchers [27,33,34] use either an end-to-end deep neural network or image features with variations of support vector machine (SVM) [35] classification algorithms to optimize food recognition. However, many studies on food recognition assume that only 1 food item is present in an image and apply a multiclass classification algorithm to identify the foods. In real-world scenarios, there is typically more than 1 food item in an image, where each food item is a segment in the image and is described by handcrafted or deep features and then classified by an SVM classifier [22,36].

##### Research Opportunities

Robust and accurate food image recognition remains a challenge because many foods have a deformable appearance and thus lack of rigid structures and because there often exists subtle differences in visual features among different food categories. Factors such as food preparation and personal preferences can also affect the appearance of food ingredients. Deep neural network–based approaches provide opportunities to improve the robustness and accuracy of food recognition systems but depend heavily on well-constructed training datasets and proper selections of neural network architectures. However, there is an opportunity to provide contextual information in the food recognition algorithm, which can include environmental cues and previous diet history. There have been advances in restaurant-specific food recognition [23,37,38] where location and menu information are used to assist with recognition. Others [39] have integrated recipe and cuisine as context and prior knowledge to aid automatic food recognition. Food patterns across time and dietary preferences are increasingly being shown to improve food classification accuracy [40]. Incorporating contextual cues can be essential to advancing the robustness of food recognition algorithms.

#### Challenge: Inaccurate Portion Size Estimation in Image

Estimating food portion size from an image is challenging as preparation and consumption impose large variations on food shape and appearance. Several food portion estimation techniques based on reconstructing the 3-dimensional (3D) models of the foods have been developed, which require users to take multiple images or videos or to modify mobile devices [41-45] to enable reconstruction. These approaches work well for irregularly shaped foods, but they do not work well when there are no strongly matched features (ie, corresponding sets of points) occurring on multiple frames. These approaches also require users to capture multiple images from different angles, making them tedious and unsuitable for long-term health monitoring and data collection. Others have focused on developing methods to estimate food portion size from a single-view image [17,46-49]. These methods use geometric model–based techniques that require food labels and food segmentation masks (ie, pixel location of foods in the image). Errors from automatic food classification and image segmentation can propagate into the final portion estimation. In addition, existing methods have only examined small model libraries consisting of foods with simple geometric shapes (eg, apples, burgers, and pizza). Further research is required to develop more comprehensive model libraries capable of dealing with irregularly shaped foods.

##### Research Opportunities

Despite some promising results from existing approaches, the performance of current portion estimation methods is not yet satisfactory. More recently, several groups [27,50] have developed portion estimation methods using deep learning. However, these techniques estimate food volumes rather than food energy. With food volumes estimated, food density is still required to compute weights, which can then be mapped to food energy using a food composition resource, such as the FNDDS [18]. Therefore, new approaches [16] that can directly link food images to food energy in the image are desirable.

Depth sensors and dual camera configurations are quickly gaining popularity on consumer mobile devices. More 3D information can be collected without significantly adding to a user’s burden capturing the eating scene. For example, mobile phones equipped with depth sensors enable simultaneous capture of image depth and the RGB color model image. For dual camera systems, at least two images are captured from slightly different angles, enabling multiview 3D reconstruction techniques. The additional information captured by the mobile devices may improve the accuracy of food portion estimation by providing additional 3D information on food objects.

### Eating Action Unit–Based Sensing

Although existing imaging technologies have shown reasonable success in estimating calorie intake and nutrients from images, there is growing interest in capturing proxies to calorie intake with sensor modalities that have fewer wearer privacy concerns. EAUs (ie, fine-grained activity units that occur during eating) are a mechanism to understand calorie intake patterns and behaviors. Accelerometer- and gyroscope-based inertial measurement units [51,52] are examples of EAUs that assess eating patterns. These techniques have been developed as a result of observing feeding gestures (or bites) and their correlation to calorie intake [53,54]. The underlying assumption of EAUs is that by counting the number of bites and estimating average calories per bite, we can provide a reasonable estimate of overall calories consumed, map the number of bites to calories to determine over- or underconsumption, and enable users to automatically quantify their calorie intake using EAU-based devices [55].

Capturing EAUs enables actionable insight, where information generated can be used by wearers, clinicians, and dietitians in a timely manner. They also enable interventionists to test the efficacy of calorie-informed, just-in-time interventions in close proximity to eating episodes. Although the detection of EAUs has shown promise, several challenges prevent these systems from being adopted in clinical and population settings (Table 2).

**Table 2:** Challenges and research opportunities in adopting eating action unit–based sensing methods.

Challenge	Research opportunity
Limited understanding of context surrounding system success and failures	Use wearable video cameras to validate contextual information surrounding when sensor–algorithm pairings fail in real-world settings
Privacy protection in *ground truth* data collection methods	Identify novel ways of protecting bystanders and other sensitive information in the field of view of cameras both in hardware and software to ensure wearer privacy concerns are addressed, thereby increasing likelihood of capturing naturally occurring behavior
Inability to accurately distinguish between food categories	Define food categories that are most useful for clinicians and researchers for diet interventions and food recalls

#### Challenge: Insufficient Understanding of the Context of System Success and Failure

Several systems have shown promise in free-living populations but fall short of delineating the contexts for when and where their systems succeed and fail, which prevents others from building on previous work to advance EAU systems under challenging scenarios. For example, gesture EAUs are confounded by smoking action units; however, few studies attempt to consider other challenging contextual scenarios that can confound eating behaviors [56]. Attempts to advance eating detection while considering challenging contexts are limited primarily because confounding contexts are not clearly delineated. A preliminary study in 8 participants who wore a wrist-worn sensor for a few hours in a free-living situation demonstrated that wearable video cameras have an approximately 38% false-discovery rate, which typically corresponded to phone-related gestures [57]. The false-discovery rate could be mitigated by integrating phone usage information with the system. However, data are limited in the context in which 1 device outperforms the other, limiting our ability to advance EAU-based eating detection systems.

##### Research Opportunity

Additional data are needed on the strengths and limitations that lead systems to succeed and/or fail in free-living populations. Understanding the context of success or failure enables identification of strengths and weaknesses of various systems and advances both the hardware and algorithm used to solve these challenges. Studies are beginning to identify the context in which sensor-algorithm pairings fail; however, they lack validity through visual confirmation. With the exception of a few recent studies [57-62], few researchers have incorporated wearable video cameras in the field to provide such validation. Researchers should continue using wearable video cameras in free-living populations and clearly state the context within which the system succeeds or fails.

#### Challenge: Inability to Protect Privacy When Using Wearable Video Cameras in the Field

Evaluation of an eating detection system necessitates testing against visually confirmed (with video) ground truth (ie, means of validating the activity in a real-world setting). Visual confirmation of eating behaviors is the strongest form of ground truth available for EAUs but also one of the most burdensome on the participant and researcher. Many researchers limit their studies to controlled settings primarily because of the limited robustness of the sensor and the time required to manually label video streams to produce ground truth. There is significant time and cost associated with designing a fail-safe device that can function in free-living environments [63]. However, people are generally unwilling to wear wearable video cameras in real-world settings owing to privacy concerns, and the likelihood of behaving naturally in the presence of a video camera is limited [64]. Recent research has shown that, given acceptable levels of incentives and properly orienting the camera, participants are willing to don wearable video cameras in real-world settings for a short period of time. Some researchers bypass the use of wearable video cameras by combining other sources of information, such as food journaling and sensor data [65], commercial electroencephalography (EEG) sensors to produce labels for a wearable EEG device [66], and a GoPro Hero 3 camera mounted on the chest facing the jaw [58].

##### Research Opportunity

Ensuring a long-lived, minimally intrusive method for capturing ground truth is necessary for capturing realistic data and rigorously testing interventions. As many participants report concerns for bystanders as their main reason for being unwilling to wear a camera all day [64,67,68], researchers may be able to design video cameras that are both privacy-preserving and that aid in validating other body-worn sensors by providing video confirmation of EAUs. Importantly, this will ensure that sensors being deployed adequately validate the behaviors they claim to capture in the settings they are most needed.

#### Challenge: Inability to Accurately Distinguish Between Food Categories

Similar to image processing, EAU-based sensors have shown success in distinguishing between different food categories. Chewing crunchy chips compared with chewing a banana produces very different sensor signals, and researchers are beginning to capitalize on these variations to distinguish between food items and type. Automatically determining solid versus liquid ingestion has provided some utility in identifying sources of ingestion behavior and intervention design, allowing researchers to investigate this phenomenon [69-71]. This could provide utility in a hospital setting, where foods provided to patients are known a priori, narrowing the food search space. However, this limits utility in free-living populations, and the challenge remains in increasing external system validity.

##### Research Opportunity

Knowledge of an individual’s diet may narrow the search space, enabling sensors to automatically distinguish between individuals, given their known diet and food environment. An opportunity exists to categorize foods in a way that would be most useful for researchers and clinicians in improving calorie intake estimates. Although distinguishing between liquid and solid consumption may have research utility, it is unknown what other types of food categories may be distinguishable. Within-subject variability of nutrients is influenced by gender, age, and education [72]. Thus, this research opportunity would be most helpful for populations with low-variability diets, particularly among elderly participants or patients on restricted diets.

### Biochemical Measure–Based Sensing

There are concerted efforts underway to characterize the biochemical changes in the body that result from food and calorie intake. Novel classes of biochemical and electrochemical sensing systems could be used to analyze changes in metabolic activity observed in interstitial fluid, saliva, or sweat [73,74]. In this section, we have reviewed wearable sweat monitoring systems that have been deployed recently in remote environments (Table 3).

**Table 3:** Challenges and research opportunities in adopting biochemical measure–based sensing methods.

Challenge	Research opportunity
On-body biochemical monitoring	Apply wearable biochemical sensors to monitor electrolytes, metabolites, and proteins in biological fluids (eg, saliva, sweat, and interstitial fluid)
Stability of wearable sensors under different environmental conditions for metabolites, electrolytes, and proteins	Develop stable biochemical tests to determine concentrations (bioassays) of glucose, lactate, cortisol, ammonium, sodium, chloride, and potassium, which require limited handling and refrigeration with dehydration or freeze-drying methods
Reusable vs single-use wearable sensors	Develop low-cost battery and energy harvesting solutions to enable single-use and multiuse modes of operation

#### Challenge: On-Body Biochemical Monitoring

Recent efforts have focused on biochemical analysis of eccrine sweat using wearable devices [75-77], which leverage both colorimetric and electronic-based sensors that collect sweat directly from skin pores and measure biomarker concentrations and dynamics (eg, sweat loss and sweat rate) in real time. This opens new possibilities for characterizing electrolyte and metabolite loss during daily activities, which can be correlated with blood metabolites, hormone, proteins, pathogens, and drugs [78,79].

Continuously monitoring biomarkers in sweat requires highly sensitive techniques for extraction and electrochemical analysis. Researchers have devised strategies to reduce the contamination effects of skin in contact with the device, while increasing sweat collection volume. This new class of wearable biochemical sensors could provide viable pathways for creating noninvasive and remote analysis of diet, wellness, and health [75]. However, shelf-life stability of biochemical sensors, susceptibility to contamination, and fundamental limitations in capturing sufficient volumes of sweat remain problematic.

##### Research Opportunity

Key opportunities lie in the design and deployment of biochemical-sensing devices that can endure temperature changes owing to environmental factors and mechanical impact while maintaining signal quality without degradation over time. Beyond device resiliency, comparisons of sweat and blood analyte levels must be tested across healthy and sick populations to determine validity and applicability of on-body sweat sensing.

#### Challenge: Enabling Robust Onboard Enzymatic and Chemical Assays (Biochemical Tests and Assays) Under All Environmental Conditions

The rich heterogeneous blend of electrolytes, metabolites, and proteins in sweat represents a unique set of noninvasively collected data. These biomarkers have been shown to correspond to the physiologic state and may serve as the basis for understanding cognitive impairment in the field. To date, most studies have focused on characterizing electrolytes and metabolites (eg, glucose or lactate) using bioassays in controlled laboratory settings. Metabolic biomarkers could change with physical stress and diet during daily activity outside of controlled laboratory settings. The stability of wearable biochemical sensors is thus crucial to maintain over extended time periods in real-world settings.

##### Research Opportunity

Wearable biochemical sensors that employ onboard dehydrating reagents or buffers that reduce degradation could lead to broad-scale deployment of these systems. Refrigeration is useful in protecting against bioassay degradation, but it requires special instructions and specialized equipment for proper handling and modes of operation. The development of new classes of wearable devices that require limited handling and refrigeration and that can handle enzyme-linked immunosorbent assay and protein-based analysis, using dehydration and freeze-drying steps to promote chemical stability, represents an area of enormous potential for robust remote-based deployment of wearable technologies.

#### Challenge: Reusable Versus Single-Use Sensors

Continuous monitoring of sweat biomarkers requires flexible electronics modules, memory storage, and onboard batteries to facilitate data capture, signal processing, and transmission. Significant practical considerations, such as sensor corrosion at the interface with ionic fluids, need for cleaning, and the resulting signal degradation that could occur over time limit the utility of reusable systems. Electrochemical sensing systems consisting of a reusable electronics module and single-use electrochemical sensors provide compelling routes to address these challenges. Single-use system designs may circumvent the challenges of long-term wear, fluid–device interface, and signal degradation. However, disposable devices must be carefully engineered to support sufficiently reduced cost to warrant single-use deployment.

##### Research Opportunity

Sweat is a corrosive biofluid that engenders significant device cleaning to facilitate reuse of the device. Thus, single-use wearable biochemical-sensing systems address important limitations of reusable systems assuming cost constraints are met. Hybrid designs, in which the reusable module mechanically couples to a single-use biochemical sensor, may mitigate the limitations of reusable and single-use systems. Although hybrid systems tend to cost more, they have significantly greater signal processing and battery capacity for long-term continuous monitoring.

### Challenges Across Sensing Modalities

Regardless of the type of sensing modality, calorie estimation techniques share a set of common challenges because of the unique role that eating and nutrition play in everyone’s lives. These challenges are related to sensor development, validation, and refinement both in controlled and free-living settings.

#### Challenge: Burden of Multimodal Systems

Although many researchers have studied detecting eating using a single wearable device, several are beginning to combine multiple sensors and context via multiple wearable devices to advance the total accuracy of an eating detection system. Mirtchouk et al [80] showed that using in-ear audio with head and wrist sensors improved accuracy from 67.8% with audio alone to 82.7% and 76.2% for head and wrist sensors, respectively. However, these approaches were mainly tested in a laboratory setting, not in a free-living environment, and it is unknown how well the findings translate to free-living populations. Multimodal sensor studies that attempt to determine utility of sensors in real-world settings are sorely needed.

Wrist sensors coupled with other sensor modalities (eg, GPS and respiratory plethysmography) may aid in distinguishing among smoking, eating, and other activities. Examples of multimodal systems include using a jaw motion sensor, a hand gesture sensor, and an accelerometer [81] and using an airflow sensor, a respiratory plethysmography chest sensor, and a wrist-worn sensor [82]. However, the burden of wearing all these sensors is significant. Thus, novel ways of combining less burdensome sensors and devices or integrating noncontact or noninvasive devices are needed while advancing the accuracy in detecting calorie and macronutrient intake or proxies.

#### Challenge: Time Asynchronization Across Sensors

A multidevice system brings challenges in coordinating and synchronizing activities and sensing across devices. As each device manages its own internal clock, this network of clocks can become unsynchronized following power failure or reset. Most devices are designed to be standalone and use an internal clock, as opposed to time stamping their data using a nearby smartphone or body sensor unit. Time synchronization in real time has been a long-studied problem; however, automated time-synchronization methods post data collection can enable researchers to test multiple devices simultaneously, without the need to reengineer the device to use a central hub. Without reliable millisecond time-synchronization techniques, annotations from 1 sensor stream (eg, video camera) are not transferable to another sensor stream.

#### Challenge: Lack of Automation in Data Labeling

Once a sensor is deployed in a real-world setting, a supervised learning model, which aims to categorize data from prior known labels or information (ie, supervised training), is designed to process the data and determine system viability. However, one challenge in building a supervised learning model is providing sufficiently annotated instances or labels to train the model. Prior studies in real-world settings have depended on self-reported annotations [59,83-86], which are burdensome and rarely timely. More recently, studies are using wearable cameras worn by participants to provide annotations through visual activity confirmation poststudy [57-62]. To visually confirm, a data labeler is hired to watch the video and label points in time when the activity occurred, which is time-consuming and prone to error. Computer scientists are beginning to design tools to automatically annotate using active learning systems that attempt to reduce the required number of annotations to build a reliable machine learning model. However, systems currently developed focus on building models that process data with samples that are fixed in time (eg, an image or a minute of data). Active learning systems designed to handle activities with varied durations (eg, eating episodes, feeding gestures, and chewing duration) can fill this gap.

#### Challenge: Unknown Acceptability of System by Users

For a system to succeed in real-world settings, it must be acceptable for the population of interest. Although several surveys have been designed to assess wearability of systems, there is no validated standard survey or approach to assess willingness to wear and use a device in the nutrition context. Current systems deploy devices for a variable amount of time (eg, 1–2 days, 1 week, or 1 month) and then report comfort based on a Likert scale. Habits regarding technology adoption are not properly understood until at least 1 week (when most individuals stop using an app or a device) [87]. As a result, acceptability must be clearly limited to the number of days the system was actually tested in free-living populations. An important contributor to system acceptability is battery lifetime, which is closely tied to device burden (ie, frequency of recharge).

#### Challenge: Short System Battery Lifetime

Long battery lifetime is essential for wearable technology to ensure high sensor sensitivity and recall of eating episodes in free-living populations. It is reported to be the most important feature rated by mobile device users [88-90]. Battery lifetime becomes critically important in longitudinal studies where reducing user burden is key to gathering more data and encouraging habituation. If users must recharge a device multiple times a day, this will limit data collection. Moreover, battery lifetime enhancements enable populations who may otherwise not be able to manage a device (eg, pediatric or geriatric populations). There are several software approaches to increase battery lifetime including duty-cycling, high-powered sensors, or triggering with low-power sensors. Reducing computational complexity and designing for specificity also reduces wasted energy. New materials enable batteryless sensing devices powered by energy harvested from the environment, wearer motion, or Wi-Fi gateways. Although these sensors show promise, they are not without challenges, as reliability can be an issue when ambient energy is not readily available. Wearable sensors are increasingly being developed to last several months [91,92], but most commercial sensors las*t* <12 hours [93] when attempting to collect continuous inertial measurement unit data. Low-maintenance sensor solutions must be designed, and careful consideration of battery lifetime must exist in every phase of system and study design.

#### Challenge: Limited Rigor of System Testing

Although technology development serves as an important contribution to the health community, reproducibility of the results is essential to determine proper construct validity, internal reliability, and test-retest reliability to increase confidence in the potential of a system to work in real-world settings. Most existing wearable sensors and systems show success by their principal investigator but have not extended beyond the laboratories in which they were implemented. To prevent bias in reporting, researchers need to disseminate their systems (hardware, software, and datasets) to other teams to provide independent testing and review. Such rigor in testing of sensing platforms is needed across all sensor modalities.

## How Technology-Enabled Devices Can Assist Conventional Subjective Diet Assessment

Conventional diet assessments comprise subjective and objective (eg, double-labeled water and metabolic chamber) approaches [94]. Although both approaches measure calorie and macronutrient intake, subjective diet assessments are more commonly implemented in research and clinical settings [95], in part owing to greater convenience and reduced cost [96]. Subjective diet assessments are not constrained by battery lifetimes and are acceptable for target populations. However, each type of subjective diet assessment introduces unique types of measurement error depending on how the diet data are being collected. A description of each assessment, and its strengths and limitations, is presented in Table 4. We posit that technology—such as image sensing, EAU, and biochemical measures—can assist subjective diet assessments to capture habitual dietary intake and eating behaviors, as systematic and random errors associated with both approaches are fundamentally independent. Replacing subjective diet assessments with technology may be difficult as other limitations could arise, such as ambiguity in identifying food images through image distortion or uncertainty over whether the food was truly consumed [97]. Combining information from technology and subjective diet assessments improves the validity of dietary intake data because the 2 approaches complement each other’s strengths and limitations.

**Table 4:** Conventional subjective measurements of energy and macronutrient intake.

Method	Description	Strengths	Limitations
24-hour diet recalls	Inquiry about everything one had to eat and drink during the previous day (usually midnight to midnight); probes often used to collect more detail and standardize the interview	Open-ended, enabling greater detail about intake and food preparation; good for culturally diverse diets; less burdensome	Memory dependent; error prone in quantifying portion sizes; requires intensive interviewer effort, which can decrease motivation to collect accurate data; repeated measures needed to capture usual intake; can alter eating behaviors if recalls are scheduled in advance
Food records	Detailed list of all foods and drinks consumed over a specified amount of time, written by respondent and ideally using weight scales or measuring tools to determine portion size; provides data about actual intake	Open-ended; does not rely on memory if records are completed on time; allows for self-monitoring	Requires intensive respondent effort, which can decrease motivation to collect accurate data or lead to poor response rate; burdensome on staff to analyze data owing to entering and coding items; repeated measures needed to capture usual intake; can alter eating behaviors since respondents are monitoring their diets
Food frequency questionnaire	Questionnaire asking whether a food item was consumed during a specified period of time; contains 2 components (food list and frequency response question); provides data about relative intake	Measures usual intake; less burdensome on respondent and research staff	Memory dependent; food list is fixed and may not capture usual intake, particularly in a culturally diverse diet; may be difficult to quantify food portions without food images; difficult to inquire about mixed dishes; respondent may have difficulty interpreting the questions

One primary concern for using subjective diet assessments includes intentional or unintentional misreporting of dietary intake [98]. Specifically, 24-hour dietary recalls and FFQs rely on memory, which can depend on age, education, attention during eating, and consistency of diet patterns [94,99]. Many individuals underestimate portion sizes for foods and beverages [100] and are sometimes provided with household items, food scales, and/or 2-dimensional images of foods with anchors to improve portion size accuracy [94]. However, these instruments create additional burden and decrease motivation to accurately capture caloric and macronutrient intake. These limitations have far-reaching implications as investigators would be uncertain if the subjective diet assessments are accurately characterizing true dietary intake [101], correctly identifying whether participants are adhering to specific diet interventions [102], and introducing bias when investigating diet-disease associations [101]. Image-sensing and EAU behavior approaches can minimize misreporting by objectively capturing food images and by identifying timing of eating episodes, allowing individuals and researchers to corroborate information from subjective diet assessments. Furthermore, image-sensing and EAU measures can also serve as visual or verbal cues to assist recall when conducting subjective diet assessments.

A limitation specific to 24-hour dietary recall and food records is the measurement of acute intake and not the usual diet [94]. Multiple measures are needed to capture usual intake, and data collection must occur for every day of the week [94]. However, increasing the number of subjective diet assessments creates greater burden for the individual and the research team. Image-sensing and EAU measures can reduce the number of 24-hour dietary recalls and food records needed to capture the best estimates of absolute dietary intake, while automating the analysis of dietary data. To this end, more research is needed to evaluate the number of images and EAU measures needed to provide the best approximation of absolute dietary intakes.

Biochemical measures can also determine nutrient status of the body. However, biochemical concentrations are not true markers of dietary intake and can reflect how the body absorbs, transports, metabolizes, and excretes the nutrient [103]. Therefore, biochemical measures cannot replace subjective diet assessments since it would be unclear how nutrient status is influenced by dietary intake or in vivo processes. Recent advances in statistical approaches, such as prediction models that use data from technology and conventional subjective approaches, account for measurement errors and can provide more accurate results [104,105].

Technology-enabled devices that measure calories and nutrients can also have far-reaching implications in clinical practice. A recent study reported that providers perceive health-tracking technologies as very useful when reviewing patient data, managing medical visits, and facilitating patient–provider communication [106]. A growing number of patients are also engaging with health technology. According to the National Cancer Institute’s 2017 Health Information Trends Survey, 34% reported owning an electronic monitoring device to track their health behaviors [107]. A growing opportunity remains in developing efficient strategies to merge technology with subjective diet assessments toward obesity prevention and treatment efforts.

## Discussion and Conclusions

Sensors from the 3 domains presented (image-based, EAU-based, and biochemical measure–based) have the potential to identify markers that improve estimates of calorie intake. However, the technologies still require considerable user input from the end user, scientist, or clinician who may have to label or segment images or metrics from such wearables to train a machine learning system. Fully automating technology-enabled calorie and nutrient monitoring would open the possibility to providing highly informed and validated information to augment recall methods and advance estimates of calorie intake for clinicians and patients.

Wearable-based sensing modalities focused on biochemical processes offer a solution for understanding food nutrients. However, more technical expertise is needed to merge conformal, low battery, secure, and valid technology with appropriate caloric assumptions. Once a stronger correlation can be drawn between biochemical products analyzed and calories consumed, biochemical-based wearables may provide promise in future automated calorie estimation systems. However, as with most wearable technologies, adherence to wearing the device remains problematic. This may be overcome if the value in such technologies pans out.

Although calories and nutrients can be consumed and monitored, it is essential to understand the behavioral choices that drive these decisions and if those behaviors can provide insight into calorie intake. All 3 systems have the potential to provide such information from cortisol levels for stressful eating (using biochemical sensors) to late-night snacking (using image-, physical-, and behavioral-based sensors) and beyond. The biggest challenge then becomes how to use the reliable big data collected from these devices to drive an actionable outcome such as lower calorie consumption or identification of eating behaviors that increase calorie intake.

It is our view that the defined research opportunities regarding calorie intake monitoring apps are the most promising, which may move the science toward a ubiquitous future of such monitoring. Nonetheless, challenges remain to fully introduce such solutions to have the desired health impact that clinicians and patients alike expect. At present, the next logical step is for scientists to improve the functionality of such devices, for human-computer interaction experts to improve usability, and for clinical teams and behavioral scientists to assess what information can be used to improve health behavior interventions given these new advanced technological tools. This translational, multidomain effort will demonstrate whether calorie intake monitoring enables higher quality of life and thus challenges the public health crisis of obesity.

## Appendix

Multimedia Appendix 1 List of attendees including faculty, postdoctoral researchers, and graduate students who participated in the discussions.

## Abbreviations

3D: 3-dimensional
AMT: Amazon Mechanical Turk
EAU: eating action unit
EEG: electroencephalography
FFQ: food frequency questionnaire
FNDDS: Food and Nutrient Database for Dietary Studies
mHealth: mobile health
RGB: red green blue
SVM: support vector machine
