# Supplementary material for: Counting Bites With Bits: Expert Workshop Addressing Calorie and Macronutrient Intake Monitoring
Source: J Med Internet Res. 2019 Dec 4;21(12):e14904. doi: 10.2196/14904 (PMC6920913; doi:10.2196/14904)
Supplement: Multimedia Appendix 1 [file jmir_v21i12e14904_app1.docx]

Multimedia Appendix 1

List of attendees including faculty, postdoctoral researchers, and graduate students who participated in the discussions**.**

| - Nabil Alshurafa, PhD - Bonnie Spring, PhD - Lisa Neff, MD - Kevin Hall, MD, PhD - Marilyn Cornelius, PhD - Adam Hoover, PhD - Edward Delp, PhD - Mingui Sun, PhD - Roozbeh Ghaffari, PhD - John Rogers, PhD - Veena Misra, PhD - Adam Hauke, PhD - Andrew Jajack, PhD - Jason Heikenfeld, PhD - Madhu Reddy, PhD - Aggelos Katsagelos, PhD - Tammy Stump, PhD - Angela Pfammatter, PhD - Dzung Nguyen, PhD - Solomon Abiola, PhD - Rawan Alharbi - Zachary King - Shibo Zhang - Ada Ng - Runsheng Xu - Robert Bell - Lida Zhang - Suman Maroju - Sara Hoffman - Gwen Ledford |
| --- |
